# Biomineralized MnO_2_ Nanoparticle-Constituted Hydrogels Promote Spinal Cord Injury Repair by Modulating Redox Microenvironment and Inhibiting Ferroptosis

**DOI:** 10.3390/pharmaceutics16081057

**Published:** 2024-08-12

**Authors:** Yuyu Sun, Jinlong Zhang, Yong Gu, Tianqing Liu, Liang Chen

**Affiliations:** 1 Department of Orthopaedic Surgery, The First Affiliated Hospital of Soochow University, 899 Pinghai Rd, Suzhou 215031, China; sunyuyunt@126.com (Y.S.); jinlongyv20120820@hotmail.com (J.Z.);; 2 Department of Orthopedic, Nantong Third People’s Hospital of Nantong University and Affiliated Nantong Hospital 3 of Nantong University, 60 Qingnian Rd, Nantong 226001, China; 3 Department of Spine Surgery, Nantong City No.1 People’s Hospital and The Affiliated Hospital 2 of Nantong University, 666 Shengli Rd, Nantong 226014, China; 4 NICM Health Research Institute, Western Sydney University, Westmead, NSW 2145, Australia

**Keywords:** manganese dioxide nanoparticles, reactive oxygen species, human apoferritin, spinal cord injury, hydrogel

## Abstract

Spinal cord injury (SCI) is one of the most severe injuries, characterized by multiple positive feedback regulatory signaling networks formed by oxidative stress and inflammation in the injury microenvironment, leading to neuronal cell damage and even death. Here, astragaloside IV (AS), known for its regulatory role in ferroptosis, was encapsulated in the cavity of apoferritin (HFn) after an in situ biomineralization process involving MnO_2_, resulting in the synthesis of HFn@MnO_2_/AS nanoparticles. These nanoparticles were then dispersed in chitosan/polyvinyl alcohol/glutaraldehyde/sodium β-glycerophosphate (CGPG) hydrogels to form CGPG-HFn@MnO_2_/AS injectable thermosensitive hydrogels that can scavenge reactive oxygen species (ROS) in the microenvironment. Our findings indicated that the prepared CGPG-HFn@MnO_2_/AS hydrogel exhibited remarkable efficacy in scavenging ROS in vitro, effectively ameliorating the oxidative stress microenvironment post-SCI. Furthermore, it inhibited oxidative stress-induced ferroptosis in vitro and in vivo by regulating SIRT1 signaling, thereby promoting neuronal cell migration and repair. Hence, the developed hydrogel combining MnO_2_ and AS exhibited multifaceted abilities to modulate the pathological microenvironment, providing a promising therapeutic strategy for central nervous system (CNS) diseases.

## 1. Introduction

Spinal cord injury (SCI) represents an acute traumatic disorder of the central nervous system (CNS) arising from incidents such as occupational injuries and traffic accidents, resulting in persistent motor or autonomic dysfunction for affected individuals [1]. The escalating prevalence of spinal cord injuries, driven in part by the surge in modern transportation, imposes a substantial societal and familial burden [2]. Globally, approximately 27 million individuals with SCI encounter enduring paralysis and depression [3]. The severely limited intrinsic regenerative capabilities of spinal cord tissues, coupled with their pronounced vulnerability and intricate pathological alterations, present formidable impediments to effective tissue repair [4]. Current clinical interventions encompass surgical procedures, pharmacotherapy, transplantation, gene therapy, and physical rehabilitation [5]. Despite the array of available approaches, prevailing clinical treatments primarily address symptoms and fall short of achieving substantial functional restoration [6]. Consequently, individuals with SCI commonly endure irreversible sensory and motor deficits and may even succumb to life-threatening complications.

Secondary injury manifests minutes to weeks after the primary injury and is intricately linked with the activation of microglia and neuroinflammation, culminating in the liberation of reactive oxygen species (ROS) and pro-inflammatory cytokines [7]. The ensuing ROS-mediated impairment of cell membrane lipids, proteins, and DNA has a profound impact on essential cellular functions, instigating a cascade of degenerative processes that induce upregulation of inflammatory and apoptotic factors. This, in turn, precipitates further demise of spinal cord tissue [8]. Post-injury, compromised mitochondria exert an adverse influence on the metabolic functions of spinal cord cells and tissues, simultaneously serving as a reservoir for ROS, thereby engendering a deleterious self-perpetuating cycle of spinal cord tissue injury [9]. The excessive production of ROS within the microenvironment is identified as a principal instigator in the establishment of an inhibitory microenvironment following spinal cord injury. This is attributed to their role in eliciting oxidative stress, cytotoxic neuroexcitation, the escalation of inflammatory responses, and the induction of ferroptosis [10,11]. Hence, the development of an efficacious strategy for mitigating ROS within the SCI microenvironment is imperative, holding significant promise for enhancing the overall efficacy of SCI treatment.

Nano-enzymes, with their nanoscale size and enzyme mimetic properties, have been widely used as substitutes for natural enzymes in a variety of applications such as biosensing, antimicrobial applications, antioxidant interventions, disease treatment, and environmental protection. Notably, within this category, manganese dioxide (MnO_2_) nano-enzymes have attracted much attention as a unique antioxidant. MnO_2_ nano-enzymes exhibit catalytic abilities similar to superoxide dismutase (SOD) and catalase (CAT). The synergistic cascade catalytic reaction of these two enzymes contributes to the effective scavenging of reactive oxygen species (ROS) in the affected region. MnO_2_ nan-enzymes exhibit faster reaction kinetics and long-lasting efficacy compared to conventional antioxidants, thus highlighting their great potential in antioxidant applications. Gao et al. [3] fabricated a hydrogel by incorporating manganese dioxide nanoparticles into hyaluronic acid to mitigate the oxidative milieu. This strategic integration demonstrated a notable enhancement in the viability of mesenchymal stem cells (MSCs). Zhu et al. [12] engineered MnO_2_ nanoplatforms that leveraged the nano-enzymatic properties of MnO_2_ to catalyze endogenous hydrogen peroxide (H_2_O_2_) within tumors. This catalytic process resulted in the generation of oxygen (O_2_), thereby augmenting the therapeutic efficacy of photodynamic therapy (PDT) for tumor treatment. These investigations provide empirical support for the commendable antioxidant capabilities exhibited by MnO_2_ when employed in a nano-enzyme capacity.

After SCI, iron overload activates reactive oxygen species generation, dysregulation of glutathione/glutathione peroxidase 4 (GSH/GPX4) metabolism, and accumulation of lipid peroxides, leading to lipid membrane degeneration and ferroptosis. Concurrently, a growing body of empirical evidence posits ferroptosis as a pivotal target in spinal cord injury (SCI) treatment [13]. Modulating the expression of ferroptosis-related proteins has been demonstrated to mitigate neuronal injury and facilitate the recovery of neurological function following SCI [14]. Consequently, an imperative has arisen for the development of an efficacious pharmaceutical intervention capable of ameliorating ROS, inhibiting ferroptosis, and ultimately alleviating the symptomatic manifestations of SCI. Astragaloside IV (AS), an efficacious constituent of Astragalus membranaceus, has exhibited a salutary impact on mitigating ROS release, oxidative stress, apoptosis, and inflammatory responses [15]. Both in vivo and in vitro experiments have corroborated AS’s capacity to attenuate lipid deposition and reduce the expression of ferroptosis-related factors [16]. In light of these findings, it is plausible to assert that AS and its formulations hold promise for potential application in the therapeutic landscape of SCI.

In recent years, significant efforts in SCI treatment have centered on the application of seed cells facilitated by delivery systems, with hydrogel scaffolds demonstrating indispensable advantages. Their three-dimensional network structure and mechanical properties akin to the extracellular matrix render them particularly effective [3]. However, conventional therapeutic approaches often require surgical implantation of hydrogels. The emergence of thermosensitive injectable hydrogels as hydrophilic carriers is garnering increasing attention due to their ability to be administered in situ. This approach circumvents the need for complex surgeries with extensive incisions, facilitates the filling of irregular gaps, enables controlled drug delivery, and provides a conduit for the co-delivery of stem cells and neurotransmitters. Therefore, the development of injectable hydrogels for the simultaneous delivery of MnO_2_ and AS represents a promising strategy in SCI treatment. Moreover, ensuring the effective penetration of drugs into damaged nerve cells is critical for optimizing therapeutic outcomes in SCI treatments. In contemporary research, apoferritin emerges as a prominently investigated carrier owing to its notable biocompatibility and biosafety [17]. Its versatile properties include: (1) an approximately 8 nm cavity structure, facilitating utilization as a nanoreactor for synthesizing inorganic nanoparticles of controlled dimensions [18,19]; (2) specific binding capability of the H-subunit (HFn) to TfR1 receptors, thereby enabling active targeted drug delivery [18,19]; and (3) robust biosafety and biocompatibility profiles. Moreover, the cavity of apoferritin accommodates enzymes, small interfering RNAs, and small-molecule therapeutic agents, thereby enhancing drug efficacy through targeted delivery. Liu et al. [16] utilized human-derived ferritin as a carrier, employing in situ biomineralization to produce uniform-sized MnO_2_ nanoparticles within its inner cavity. Notably, aberrant iron metabolism observed in spinal cord injuries leads to heightened transferrin receptor expression in neuronal cells, thereby underscoring the potential of HFn-based delivery systems to achieve targeted drug delivery and augment therapeutic outcomes.

Here, we proposed the use of recombinant apoferritin as a nanoreactor to generate MnO_2_ nano-enzymes in its cavity using biomineralization while encapsulating AS and dispersing it in CGPG, an injectable temperature-sensitive hydrogel. Thus, the oxidative stress microenvironment after SCI was alleviated, while neuronal ferroptosis was inhibited, and its repair and migration were promoted for the treatment of SCI. In this system, CGPG demonstrated temperature-sensitive injectability designed for in situ drug delivery via injection. Given the heightened expression of the TfR1 receptor on damaged neuronal cells, the dispersed HFn@MnO_2_/AS contained within the hydrogel can selectively target neuronal cells through receptor-mediated endocytosis. This targeted approach facilitates efficient drug delivery, thereby achieving therapeutic effectiveness. The MnO_2_ in the prepared CGPG-HFn@MnO_2_/AS therapeutic system could effectively scavenge endogenous H_2_O_2_ in the microenvironment of an SCI and generate O_2_ to alleviate the hypoxic microenvironment, while AS could alleviate its hypoxic microenvironment by modulating SIRT1 signaling to attenuate the iron death of neuronal cells, thus promoting the recovery of spinal cord neurological function after SCI. Consequently, the synergistic utilization of MnO_2_ and AS within prepared hydrogels offers a comprehensive modulation of pathological microenvironments, fostering neural repair and presenting a promising therapeutic strategy for central nervous system (CNS) disorders.

## 2. Materials and Methods

### 2.1. Materials

Chitosan (Mw~100,000 Da), β-Glycerol phosphate disodium (β-GP), and astragaloside IV (AS) were supplied by Solarbio Technology Co., Ltd. (Beijing, China). PVA (Mw~15,000 Da) was obtained from Dalian Diligence Trade Co., Ltd. (Dalian, China). Fetal bovine serum (FBS), RPMI 1640, and Penicillin-streptomycin were obtained from Beyotime (Shanghai, China). Glutaraldehyde (GA) was purchased from Sigma-Aldrich. Isopropyl β-D-1-thiogalactopyranoside (IPTG) was purchased from Sangon Biotechnology Co., Ltd. (Shanghai, China). The reactive oxygen species (ROS) assay kit was purchased from Beyotime (Shanghai, China).

### 2.2. Method

#### 2.2.1. Preparation and Characterization of HFn@MnO_2_/AS

##### Expression and Purification of HFn Protein

The protein was expressed and purified following a previously reported methodology [17]. Initially, Escherichia coli (*E. coli*) harboring the plasmid carrying the gene sequence for the protein of interest was cultivated in 10 mL of Luria–Bertani (LB) medium supplemented with ampicillin. Following activation, the bacterial culture was subsequently scaled up to 1000 mL in an LB-Amp+ medium for further incubation. Upon reaching an optical density at 600 nm (OD_600_) exceeding 0.8, induction of protein expression was initiated by the addition of isopropyl-β-D-thiogalactopyranoside (IPTG), followed by an additional 10 h of cultivation. After induction, cells were harvested by centrifugation at 10,000 rpm and 4 °C, and the resulting cell pellet was resuspended in an appropriate buffer. Cell disruption was achieved using a cell disruptor maintained at low temperatures, followed by centrifugation at 10,000 rpm and 4 °C for 30 min to yield a clarified supernatant. This supernatant was subjected to heat treatment at 70 °C for 20 min to precipitate impurities, and the solution was centrifuged again to eliminate proteinaceous precipitates. The resulting supernatant, enriched with the target protein, was retained. The final purification of the expressed HFn was accomplished through affinity chromatography, culminating in the isolation of the desired protein.

##### Synthesis of HFn@MnO_2_ Using Biomineralization

It has been reported that the inner cavity of apoferritin can be used as a nanoreactor to synthesize inorganic nanoparticles of uniform size [18]. Therefore, the synthesis of HFn@MnO_2_ nanoparticles was carried out according to the literature. Briefly, 200 μL of 0.1 M MnCl_2_ solution was added to HFn solution dropwise under continuous stirring at 100 rpm, and stirring was continued for another 2 h. Then, the pH of the above solution was adjusted to 11 by adding 0.5 M NaOH solution. After 2 h of maintenance, the pH of the solution was adjusted to 8.0 using 0.1 M HCl, and the solution was centrifuged at 14,000 rpm for 10 min to collect the supernatant. Finally, the solution was placed in a dialysis bag of 8000~14,000 Da, and unreacted MnCl_2_ was removed through dialysis.

##### Preparation and Characterization of HFn@MnO_2_/AS NPs

Next is the encapsulation of AS. First, the pH of the HFn@MnO_2_ nanoparticle solution was adjusted to 10 to enlarge the pores of apoferritin. Then, 200 μg of AS was pre-dissolved in dimethyl sulfoxide and slowly added to the above solution. After an overnight incubation, the pH of the solution was adjusted to 8.0 by adding 0.1 M HCl to allow the resumption of the apoprotein. The prepared HFn@MnO_2_/AS nanoparticles were placed in ultrafiltration tubes with a MWCO of 10 KDa and centrifuged three times at 1000 rpm to remove the unencapsulated AS.

Finally, we performed a relevant characterization of the prepared nanoparticles. The hydrodynamic particle sizes, zeta-potentials, and polydispersity index (PDI) of HFn and HFn@MnO_2_/AS nanoparticles were measured using a Malvern Nano ZS 90 (Malvern Instruments Ltd., Malvern, UK). The morphology of the HFn and HFn@MnO_2_/AS nanoparticles was detected using transmission electron microscopy (TEM, JEOL, Tokyo, Japan). The concentration of the encapsulated AS was measured using high-performance liquid chromatography equipped with an ELDS detector (HPLC-ELDS) and a C18 column. For AS, the mobile phase was Acetonitrile: Water (45:55, *v*/*v*). A total of 20 μL of sample solution was injected and run at a flow rate of 1.0 mL/min. The structural stability of HFn before and after the preparation of HFn@MnO_2_/AS nanoparticles was confirmed using native polyacrylamide gel electrophoresis (native PAGE) and circular dichroism (CD) spectroscopy. The particle size of HFn@MnO_2_/AS nanoparticles in a PBS solution containing 10% serum was detected using DLS after being stored at 4 °C for 7 days.

#### 2.2.2. Preparation and Characterization of CGPG Hydrogel

Pre-experiments demonstrated that the gel strength of CS/β-GP (CG) hydrogels with temperature-sensitive properties was poor. Thus, to enhance the gel strength of the hydrogels, PVA and GA were incorporated as a physical cross-linking agent and a chemical cross-linking agent to effectively control the drug release. The hydrogel was prepared as follows. First, chitosan (CS) and PVA were added to a 0.1 M acetic acid solution, respectively, and stirred continuously until completely dissolved. Then, an appropriate amount of PVA solution was added to 1 mL of CS solution and mixed evenly. The GA solution was then added with constant stirring and allowed to react for 10 min. Finally, β-GP solution was added dropwise to the above solution under ice bath conditions, and stirring was continued for 60 min to mix evenly. CS/β-GP (CG), CS/β-GP/PVA(CGP), and CS/β-GP/GA(CGG) were prepared according to the same steps as above.

Next, the properties of hydrogels were examined. First, the prepared CG, CGP, CGG as well as CGPG gel solutions were solidified sufficiently at 37 °C to form a hydrogel. Then, the hardness of the hydrogels was determined using a texture analyzer (CTX Texture Analyser, Brookfield, Berwyn, IL, USA) to determine their mechanical strength. Next, a rotational rheometer (DVNext, Brookfield, Berwyn, IL, USA) was used to measure the rheological properties of the prepared hydrogel systems. The conditions of measurement were as follows. The frequency was 1 Hz, the strain was 0.05%, the heating range was 15~45 °C, and the heating rate was 1 °C/min. The elastic modulus G′ (storage modulus) and viscous modulus G″ (energy consumption modulus) were selected as indicators. The phase transition temperature was the temperature when G′ was equal to G″. Scanning electron microscopy (SEM) was selected to observe the morphology of the CGPG hydrogels. In vitro degradation of the hydrogels was examined using the remaining weight. The hydrogel was weighed as m_0_ and placed in a PBS solution with a pH of 7.4 with continuous shaking at 100 rpm in a shaker at 37 °C. At predetermined time points, the hydrogel was taken out and weighed as m_t_. Then, the in vitro degradation of the hydrogel was calculated according to the formula: weight remaining = m_t_/m_0_ × 100%.

For drug-loaded hydrogels, the prepared HFn@MnO_2_/AS nanoparticles were dispersed in the hydrogel solution. The in vitro release of AS from the CGPG-HFn@MnO_2_/AS hydrogel was detected. The formed hydrogel was placed in PBS with a pH of 7.4 at 37 °C with a speed of 100 rpm. At scheduled time points, 1 mL of solution was collected, and an equal volume of PBS solution with a pH of 7.4 was supplemented. The collected solution was filtered through a 0.22 μm filter membrane and examined using HPLC-ELDS to calculate the released AS.

#### 2.2.3. Characterization of MnO_2_ Related Properties

##### The Degradation Behavior of HFn@MnO_2_/AS Nanoparticles

A dialysis method was selected to evaluate the in vitro degradation of MnO_2_ from HFn@MnO_2_/AS. Briefly, 5 mL of HFn@MnO_2_/AS was placed into the dialysis bag (MWCO 8000~14,000 Da) and placed in PBS with or without H_2_O_2_ (100 μM). At different time intervals, a fixed volume (1 mL) of the release solution was withdrawn and then replaced with the same buffer to keep the volume constant. The concentration of released Mn^2+^ was quantified using atomic absorption spectroscopy (AAS). The percent of retained MnO_2_ was calculated according to the concentration of released Mn^2+^.

##### The Antioxidant Ability of CGPG-HFn@MnO_2_/AS Hydrogels

The antioxidant activity of the CGPG-HFn@MnO_2_/AS hydrogel was characterized by scavenging H_2_O_2_. First, hydrogels with or without HFn@MnO_2_/AS were fabricated in a 24-well plate. Then, 1 mL of hydrogen peroxide solution (H_2_O_2_, 100 μM) was added to each well. After shaking at 50 rpm for different times, the upper solution was taken for the subsequent detection. Finally, the concentration of H_2_O_2_ was detected using a hydrogen peroxide assay kit (Beyotime, Shanghai, China).

Meanwhile, to evaluate the protective function of HFn@MnO_2_/AS NPs on cells against ROS, PC12 cells were seeded in a 24-well plate at a density of 2 × 10^4^. Then, 20 μM of H_2_O_2_ peroxidized medium was used to stimulate the cells for 1 h. The culture medium was subsequently replaced with different formulations and incubated for another 24 h. The expression of ROS in the cells was detected using a DCFH-DA staining kit (Beyotime, Shanghai, China) and observed using a laser scanning confocal microscope (CLSM, Leica TCS SP8, Wetzlar, Germany).

#### 2.2.4. Cell Culture and Treatment

PC12 cells, derived from the neural crest, belong to the adrenal pheochromocytoma cell line, have nerve cell characteristics, and can be used in the study of spinal cord neuronal injury. Briefly, PC12 cells (Cell Bank of the Chinese Academy of Sciences, Shanghai, China) were cultured in Dulbecco’s modified Eagle medium (DMEM, Coring, Manassas, VA, USA) containing 10% fetal bovine serum (Gibco, Gaithersburg, MD, USA) and 1% penicillin and streptomycin (Beyotime, Shanghai, China) in a standard environment (37 °C, 5% CO_2_). The cells were used for follow-up experiments when the density of the cells reached 70~80%. Then, the cells were seeded into a 6-well plate at a density of 1 × 10^6^/well and divided into five groups: control, H_2_O_2_, HFn@MnO_2_/AS (10 μM), CGPG, and CGPG-HFn@MnO_2_/AS (10 μM). The cells were pretreated with HFn@MnO_2_/AS, CGPG, or CGPG-HFn@MnO_2_/AS for 1 h, followed by incubation with H_2_O_2_ (200 μM) for 24 h. Finally, the cells were collected for the detection of various biochemical indicators.

##### Cell Uptake

HFn@MnO_2_/AS exhibited a sustained release pattern from the CGPG-HFn@MnO_2_/AS hydrogel. To characterize both the release kinetics and the cellular uptake proficiency of HFn@MnO_2_/AS, the fluorescence intensity within PC12 cells was examined utilizing Confocal Laser Scanning Microscopy (CLSM). In brief, PC12 cells were seeded into a 12-well plate at a density of 1.0 × 10^4^ cells/well and cultured for 24 h at 37 °C with 5% CO_2_. Subsequently, the culture medium was replaced with a hydrogel-loaded medium containing FITC-labeled HFn@MnO_2_/AS. Following incubation periods of 8, 12, and 24 h, the hydrogel was removed, and PC12 cells were washed with pre-cooled phosphate-buffered saline (PBS). After fixation with 4% paraformaldehyde for 10 min, the nuclei were stained with DAPI for an additional 10 min at room temperature. The resulting images were captured using CLSM.

##### Cell Viability Assay

The viability of PC12 cells was evaluated using the Cell Counting Kit-8 (CCK-8) assay kit (Beyotime, Shanghai, China). Briefly, the cells were seeded into a 96-well plate at a density of 1 × 10^3^/well for 24 h. Then, HFn@MnO_2_/AS, CGPG, or CGPG-HFn@MnO_2_/AS was added into the culture medium. After 1 h, H_2_O_2_ (200 μM) was added to the culture solution to stimulate the cells. After 24 h, CCK-8 solution (10 μL) was added into each well and cultured for 1.5 h at 37 °C. Finally, the optical density (OD) value at 450 nm was measured using a microplate reader (Bio-Rad, Hercules, CA, USA).

##### Cell Migration Assay

The ability of cell migration is thought to reflect the repair ability of cells and was evaluated by the wound healing assay. Briefly, the cells were seeded into a 6-well plate at a density of 1 × 10^6^/well. A sterilized pipette tip of 200 μL was used to draw a straight line right in the middle of each hole when the density of the cells reached 80%. Then, the cells were pretreated with HFn@MnO_2_/AA, CGPG, or CGPG-HFn@MnO_2_/AA, followed by an H_2_O_2_ challenge. The migration of PC12 cells was observed at 6, 12, and 24 h under a standing biological microscope (Olympus, Tokyo, Japan) after treatment with H_2_O_2_ and photographed for analysis.

##### Detection of Cellular Iron Level

The status of ferroptosis can be evaluated by the detection of iron concentration. The cellular iron level was evaluated using an iron assay kit (Elabscience, Wuhan, China). Briefly, the cells were seeded into a 6-well plate at a density of 1 × 10^6^/well. After treatment, the cells were harvested, lysed, and homogenized at 4 °C. The supernatant was collected and incubated with the chromogenic solution at 37 °C for 10 min. Finally, the optical density (OD) value at 593 nm was measured using a microplate reader (Bio-Rad, CA, USA).

##### Measurements of Antioxidant Levels

GSH, MDA, and SOD are generally considered to be the key indicators of oxidative stress and ferroptosis. The levels of GSH and MDA and the activity of SOD were detected using a GSH kit (Jiancheng Bioengineering Institute, Nanjing, China), MDA kit (Beyotime, Shanghai, China), and SOD kit (Beyotime, Shanghai, China), respectively. Briefly, the cells were seeded into a 6-well plate at a density of 1 × 10^6^/well. After treatment, the cells were harvested, lysed, and homogenized at 4 °C. The supernatant was collected and, for the specific testing process, please refer to the manufacturer’s instructions.

##### Detection of ROS

ROS production is also considered a key indicator of oxidative stress and ferroptosis. The level of ROS was measured by the reactive oxygen species assay kit (Beyotime, Shanghai, China). Briefly, the cells were seeded into a 6-well plate at a density of 1 × 10^6^/well. After treatment, the cells were incubated with DCFH-DA for 20 min at 37 °C. The cells were washed with serum-free cell culture solution to remove DCFH-DA that did not enter the cells. Finally, the cells were incubated with DAPI and observed under a fluorescence microscope (Olympus, Tokyo, Japan).

#### 2.2.5. Animals and Experimental Design

Adult Sprague-Dawley rats (SPF, male, 200~220 g) were purchased from Sipur-Bikai Laboratory Animal Co., LTD (Shanghai, China) and kept in the Center of Experimental Animals of Nantong University where the environment was in accordance with standard conditions (25 ± 2 °C, 40~70% humidity, 12 h light/dark cycle). This study was approved by the Animal Care Ethics Committee of Nantong University (approval No. IACUC202221013-1012). The rats were allowed to eat and drink freely and were acclimatized for at least 1 week before the experiments.

Fifty SD rats were randomly divided into five groups: sham, SCI, CGPG-HFn@MnO_2_, CGPG, and CGPG-HFn@MnO_2_/As (10 mg/kg). After the successful modeling of SCI, the animals were treated with the drug continuously for 8 weeks. The Basso, Beattie, and Bresnahan (BBB) locomotor rating score, footprint analysis, and HE staining were used to evaluate the locomotion recovery of rats after treatment. Western blot assays and kit testing were used to explore the mechanisms of drug treatment. For the specific reference steps for kit testing, please refer to the previous experimental process.

##### SCI Modeling

The animal model of SCI was constructed according to a previous study with some modifications [20]. Briefly, the rats were fixed on the operating table in the prone position, and 2% isoflurane was inhaled by the rats through a breathing mask to maintain anesthesia. The T10 spinous process was located, the skin was cut with surgical scissors, subcutaneous tissue, and fascia were separated, and the T9 and T10 laminae were exposed. The T9 and T10 spinous processes and laminae were then removed with a bone rongeur, exposing the white spinal cord and the dorsal central spinal vessel. Moderate contusion was applied to the exposed segment of T9 using a MASCIS Impactor (Model II, W.M.Keck Center, New York, NY, USA). The wound was cleaned with saline and sutured. Hind limb paralysis after the animal woke up from anesthesia indicated successful contusion modeling. For the Sham group, the rats were only subjected to a laminectomy.

##### Behavioral Testing

The BBB score and footprint analysis were carried out as previously described [21]. The BBB score, a 21-point scale, is widely used to evaluate the restoration of motor function in animals. The rats were placed in an open field and allowed to move freely for 5 min before formal testing, and the motor capacity of rats was observed from weeks 1 to 8 after SCI. In the footprinting experiment, rats were immersed in red and blue ink on the front and back legs, respectively, and then guided to walk straight on a piece of white paper. Finally, the footprints of the rats were photographed and analyzed.

##### H&E Staining

H&E staining is widely used in the study of histological lesions, which can clearly show the tissue structure of the lesions. After the rats were sacrificed under anesthesia, the spinal cord injury area segment (1 cm) was removed and fixed with 4% paraformaldehyde. After 24 h, the tissue was frozen and sliced at a thickness of 15 μm using a freezing microtome (Thermo Fisher Scientific Inc., Waltham, MA, USA). The sections were incubated with a hematoxylin and eosin solution. Finally, the sections were sealed with neutral resins and observed under a microscope (Olympus, Tokyo, Japan).

##### Western Blotting

Animals were anesthetized and sacrificed, and spinal cord tissue was collected. The collected tissues were lysed with RIPA lysis buffer (Beyotime, Shanghai, China). The total protein concentration was determined using a BCA protein assay kit (Beyotime, Shanghai, China). Extracted proteins (50 μg) were separated using 10% SDS-PAGE and transferred onto a polyvinylidene fluoride membrane (PVDF). The membrane was incubated with 5% (*w*/*v*) non-fat dry milk at room temperature for 2 h, followed by incubation with primary antibodies (SIRT1, XCT, GPX4, 4-HNE, TFR1, or GAPDH) for 24 h at 4 °C. These antibodies were purchased from Abcam. Then, the membrane was washed and incubated with the corresponding secondary antibodies horseradish peroxidase goat anti-rabbit IgG (Beyotime, Shanghai, China) at room temperature for 2 h. Finally, the membrane reacted with an enhanced chemiluminescence solution (ECL), and the blots were observed using an enhanced chemiluminescence system (Tanon, Shanghai, China).

#### 2.2.6. Safety Profiles

Healthy Sprague-Dawley rats were randomly allocated into three groups (n = 3). Blood biochemical analysis was performed after the administration of the CGPG-HFn@MnO_2_/AS hydrogel, and untreated healthy Sprague-Dawley rats served as the control group. Blood samples were obtained to conduct blood biochemistry and routine blood analyses aimed at assessing the safety profile of the nanoparticles. The parameters assessed included red blood cell count (RBC), hemoglobin level (HGB), platelet count (PLT), white blood cell count (WBC), blood urea nitrogen (BUN), aspartate aminotransferase (AST), alanine aminotransferase (ALT), and alkaline phosphatase (ALP).

#### 2.2.7. Statistical Analysis

The data were analyzed using GraphPad Prism 9.5 software, and the results are expressed as the mean ± SEM in triplicate. Comparisons between the two groups were performed using a one-way analysis of variance (ANOVA), followed by Tukey’s post hoc test. A *p*-value of less than 0.05 (*p* < 0.05) was considered statistically significant.

## 3. Results and Discussion

### 3.1. Preparation and Characterization of HFn@MnO_2_/AS

As reported, HFn exhibiting commendable biocompatibility has been documented not only as proficient carriers for drug delivery but also as nanoreactors enabling the controlled synthesis of inorganic nanoparticles with adjustable dimensions [18,19]. Utilizing a genetic engineering approach, we produced and expressed HFn, as outlined in the literature [18].

**Figure 1:**
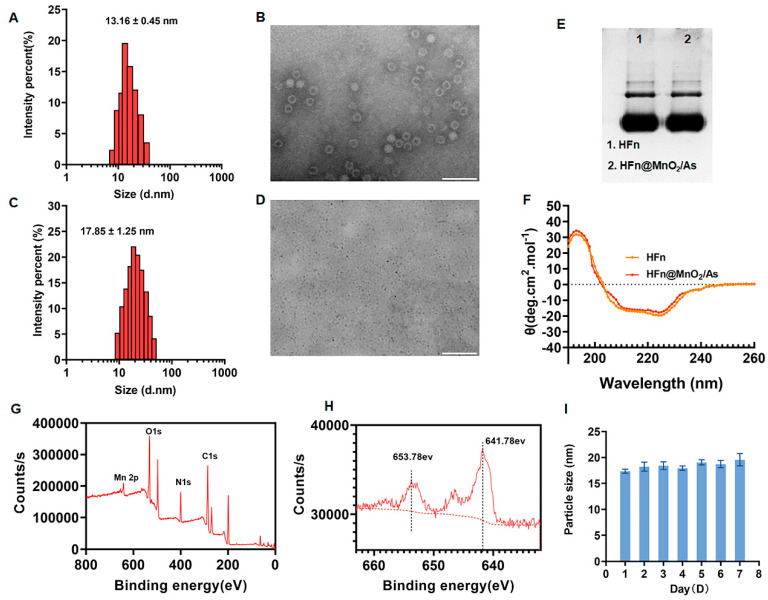
(**A**) Hydrodynamic particle size distribution of HFn detected by DLS. (**B**) The morphology image of the prepared HFn observed by TEM with negative staining by using 2% phosphotungstic acid (Scale bar: 50 nm). (**C**) Hydrodynamic particle size distribution of HFn@MnO_2_/AS nanoparticles detected by DLS. (**D**) The morphology image of HFn@MnO_2_/AS nanoparticles observed by TEM without negative staining (Scale bar: 100 nm). (**E**) Native page image of HFn and HFn@MnO_2_/AS nanoparticles. (**F**) CD spectra of HFn and HFn@MnO_2_/AS nanoparticles. (**G**) XPS analysis of HFn@MnO_2_/AS nanoparticles with full scan. (**H**) Mn 2p core-level spectra of HFn@MnO_2_/AS nanoparticles detected by XPS. (**I**) The hydrodynamic diameters of HFn@MnO_2_/AS NPs stored in PBS and PBS contained 10% FBS solutions at 4 °C for 7 days.

The particle size of the synthesized HFn was determined to be 13.16 ± 0.45 nm, with a polydispersity index (PDI) of 0.15 ± 0.08, as depicted in Figure 1A. The zeta potential of HFn was −17.34 ± 0.12 mV. The morphology of HFn was further examined through transmission electron microscopy (TEM), revealing a uniformly dispersed circ1ular structure with a hydrated particle size of 18.25 ± 0.33 nm (Figure 1B). Concurrently, HFn@MnO_2_/AS nanoparticles underwent characterization using dynamic light scattering (DLS) and TEM. The outcomes indicated an increase in particle size subsequent to the incorporation of MnO_2_ and AS into HFn, resulting in a size of 17.85 ± 1.25 nm and a PDI of 0.16 ± 0.03 (Figure 1C). The zeta potential of HFn@MnO_2_/AS nanoparticles was −15.86 ± 0.08 mV. The TEM image of HFn@MnO_2_/AS exhibited uniformly sized small black dots measuring approximately 10 nm (Figure 1D). It is noteworthy that, owing to the absence of negative phosphotungstic acid staining in the assay, the small black dots evident in the image correspond to the generated MnO_2_ particles. This observation signifies the successful synthesis of MnO_2_ employing HFn as a nanoreactor.

**Figure 2:**
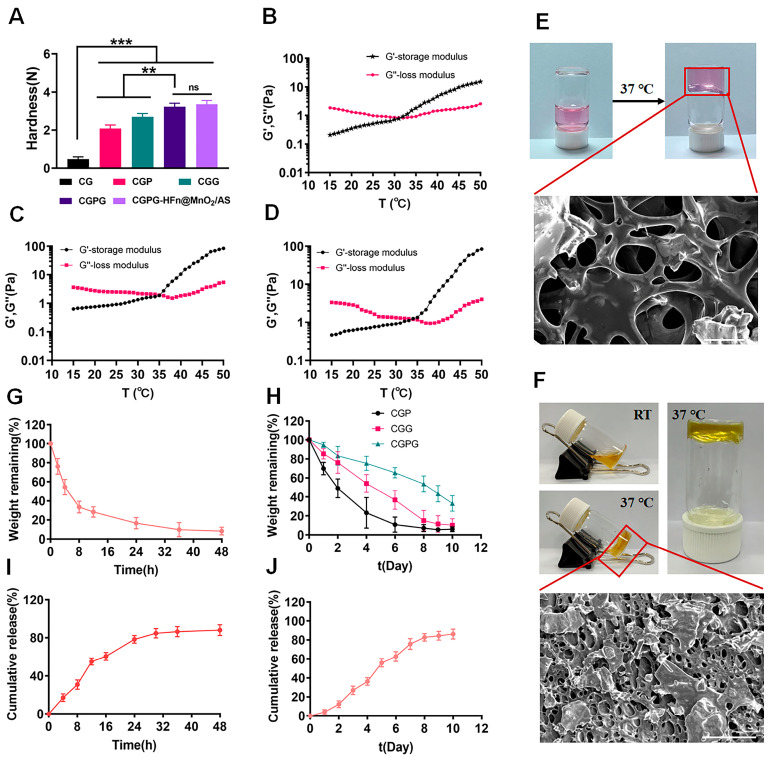
(**A**) The mechanical properties of CS, CGP, CGG, CGPG, and CGPG-HFn@MnO_2_/AS hydrogels. n = 3, ** *p* < 0.01, *** *p <* 0.001. Rheological G′ and G″ against temperatures of GC (**B**), GCPC (**C**), and GCPC-HFn@MnO_2_/AS (**D**) hydrogels. (**E**) The gel formation image of blank hydrogels at 37 °C and the SEM micrograph of blank CGPG hydrogels (Scale bar: 50 μm). (**F**) The gel formation image of CGPG-HFn@MnO_2_/AS hydrogels at 37 °C and the SEM micrograph of CGPG-HFn@MnO_2_/AS hydrogels (Scale bar: 200 μm). (**G**) The in vitro degradation analysis of GC hydrogels. (**H**) The in vitro degradation analysis of CGP, CGG, and CGPG hydrogels. (**I**) The in vitro cumulative release of AS from HFn@MnO_2_/AS nanoparticles. (**J**) The in vitro cumulative release of AS from CGPG-HFn@MnO_2_/AS hydrogels.

Subsequently, Native PAGE electrophoresis and circular dichroism (CD) spectroscopy were employed to scrutinize potential alterations in the structural morphology of HFn throughout the encapsulation process. As illustrated in Figure 2E, the HFn@MnO_2_/AS nanoparticles exhibited identical bands and positions to HFn, suggesting that the conformation of HFn remained unaltered throughout the preparation procedure. CD spectroscopy serves as a valuable tool for characterizing the secondary structure of biomolecules. As depicted in Figure 1F, the spectra revealed negative peaks at 210 nm and 220 nm wavelengths, along with a positive peak at 190 nm, indicative of the classical α-helix structure. Notably, the CD spectra plots of HFn and HFn@MnO_2_/AS nanoparticles exhibited no significant differences, implying that the nanoparticle preparation process does not induce alterations in the secondary structure of HFn.

X-ray Photoelectron Spectroscopy (XPS) analysis was employed to elucidate the surface chemistry and valence states of MnO_2_ within HFn@MnO_2_/AS nanoparticles. The XPS spectra depicted in Figure 1G exhibited the presence of carbon (C), nitrogen (N), oxygen (O), and manganese (Mn). Given the significance of metal valence states in catalytic processes, we conducted a detailed examination of the high-resolution Mn 2p XPS spectra (Figure 1H). Remarkably, the Mn 2p spectra revealed two distinctive peaks centered at 641.78 eV and 653.78 eV, corresponding to Mn 2p_3/2_ and Mn 2p_1/2_, respectively, characteristic of manganese dioxide. The observed spin-energy separation at 12 eV can be attributed to the +4 oxidation state of manganese [22]. It is widely acknowledged that elevated metal valence states contribute to the decomposition of H_2_O_2_ and the generation of O_2_. Consequently, HFn@MnO_2_/AS nanoparticles emerge as a nano-enzyme with pronounced peroxidase and catalase activities, holding promise for the effective modulation of the reactive oxygen species (ROS) microenvironment. The stability of HFn@MnO_2_/AS NPs was also evaluated (Figure 1I). The results showed that the particle size of HFn@MnO_2_/AS NPs did not change from day 0 after 1 week of storage at 4 °C, indicating good storage stability.

Conclusively, we ascertained the encapsulation rate and drug loading of AS. An amount of 6.56 ± 0.78% AS was encapsulated into HFn@MnO_2_ nanoparticles.

### 3.2. Characterization of CGPG Hydrogels

According to the reported findings, hydrogels comprising chitosan (CS) and β-glycerophosphate (β-GP) exhibit notable temperature-sensitive characteristics but demonstrate suboptimal mechanical properties, potentially resulting in drug leakage or rapid release. Consequently, the incorporation of a physical cross-linking agent (polyvinyl alcohol, PVA) or a chemical cross-linking agent (glutaraldehyde, GA) was contemplated to bolster the mechanical integrity of the hydrogel system. As depicted in Figure 2A, the introduction of PVA led to a substantial enhancement in the strength of the prepared hydrogels. This improvement can be attributed to the abundance of hydroxyl groups in PVA, facilitating the formation of hydrogen bonds with the primary amine groups on chitosan. Consequently, this interaction augmented the mechanical strength of the hydrogels. However, it is noteworthy that the stability of this physical bonding was compromised due to the reversible nature of hydrogen bonding. In contrast, the addition of GA as a chemical cross-linking agent resulted in the formation of dynamic imine bonds between the aldehyde group in GA and the amine group in chitosan. This chemical bonding conferred self-healing properties upon the hydrogel, significantly enhancing its mechanical characteristics. The combined incorporation of GA and PVA proved to be particularly effective in improving the network structure and mechanical strength of the CG hydrogels. In conclusion, the synergistic presence of GA and PVA serves as an efficient strategy for enhancing the mechanical properties of CGPG hydrogels by reinforcing the network structure [23,24]. Meanwhile, the mechanical strength of CGPG-HFn@MnO_2_/AS hydrogels after the incorporation of HFn@MnO_2_/AS nanoparticles was also determined. As shown in Figure 2A, the mechanical strength of CGPG-HFn@MnO_2_/AS hydrogel was slightly higher than that of CGPG, but there was no significant difference, which indicated that the incorporation of HFn@MnO_2_/AS nanoparticles did not change the mechanical strength of the CGPG hydrogels.

In the subsequent analysis, the storage modulus (G′) and loss modulus (G″) of hydrogels based on carboxymethyl cellulose (CG), carboxymethyl cellulose/glycerol (CGPG), and carboxymethyl cellulose/glycerol-polyvinyl alcohol-glutaraldehyde (CGPG-HFn@MnO_2_/AS) were systematically evaluated. Measurements were conducted over a temperature range of 15 to 45 °C at a constant frequency of 1 Hz, as illustrated in Figure 2B–D. The gelation temperature, denoted by the intersection of the G′ and G′’ curves, signifies a crucial transition point. When the ambient temperature fell below the gelation temperature, G″ surpassed G′, indicating a solution state. Conversely, when the temperature exceeded the gelation threshold, G″ notably decreased below G′, indicating the formation of a semi-solid hydrogel [25]. Specifically, gelation temperatures for CG and CGPG were determined to be 32.14 ± 0.16 and 35.27 ± 0.21 °C. Notably, the encapsulation of HFn@MnO_2_/AS did not alter the gelation temperature of the CGPG system. In comparison to CG, the inclusion of polyvinyl alcohol (PVA) and glutaraldehyde (GA) raised the gelation temperature of CGPG.

To explore the in situ temperature-sensitive properties, blank CGPG and CGPG-HFn@MnO_2_/AS solutions were incubated at 37 °C for 5 min to observe hydrogel formation, as depicted in Figure 2E,F. Concurrently, scanning electron microscopy (SEM) micrographs portrayed the morphology of CGPG hydrogels, revealing a dense and porous structure. This observation suggests that the combination of physical association and chemical cross-linking contributes to a continuous hydrogel network, enhancing mechanical strength [26].

In vitro degradation studies were carried out by assessing the residual weight of CG, CGP, CGG, and CGPG hydrogels in solutions. Figure 2G illustrates a rapid degradation of CG hydrogels within 48 h, with 71.66% weight loss observed in the initial 12 h. The addition of PVA and GA significantly slowed down the degradation rate, with complete degradation of CGP and CGG hydrogels occurring on days 6 and 8, respectively. In contrast, only 66.82% of the original weight of CGPG hydrogels was eroded at day 10. This aligns with the mechanical strength results, indicating that the increased strength of the CGPG hydrogel system impedes its in vitro degradation.

The distinctive three-dimensional porous structure of hydrogels positions them as excellent carriers for biologically active molecules, cells, or drugs [3]. The release behavior of astragaloside IV (AS) from CGPG-HFn@MnO_2_/AS hydrogels and HFn@MnO_2_/AS nanoparticles was evaluated to determine their sustained release capability. Figure 2I reveals that the cumulative release of AS from HFn@MnO_2_/AS nanoparticles reached approximately 85% at 30 h, whereas in CGPG-HFn@MnO_2_/AS hydrogels, around 80% of released AS was observed on day 8. This implies that the presence of CGPG hydrogels substantially hinders the release rate of encapsulated AS, preventing sudden release.

### 3.3. Characterization of MnO_2_-Related Properties

#### 3.3.1. The Degradation Behavior of HFn@MnO_2_/AS Nanoparticles

**Figure 3:**
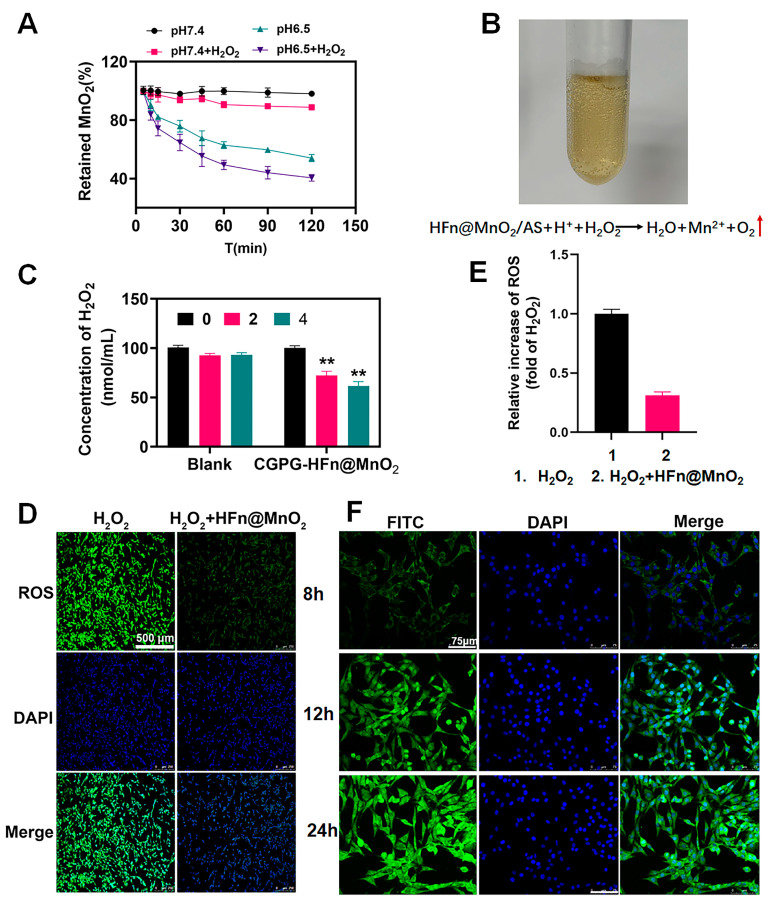
(**A**) The degradation behavior of HFn@MnO_2_ incubated in different pH solutions (pH 7.4 and 6.5) with or without H_2_O_2_. (**B**) O_2_-bubbles image observed after incubation with H_2_O_2_ in an acidic condition to confirm the formation of MnO_2_. (**C**) Evaluation of in vitro antioxidant effects of blank CGPG hydrogels and CGPG-HFn@MnO_2_/AS hydrogels in the absence of cells; ** *p <* 0.01. (**D**) Evaluation of antioxidant effects of HFn@MnO_2_/AS in PC12 cells after being stimulated by H_2_O_2_ (Scale bar: 500 μm) (**E**) Analysis of the ROS fluorescence intensity according to section D by Image J (version v1.53t). (**F**) Cellular uptake of FITC-HFn@MnO_2_/AS in CGPG hydrogels by PC12 cells detected by CLSM after incubation for 8, 12, and 24 h. (Scale bar: 75 μm).

The degradation of MnO_2_ was assessed through the quantification of released Mn^2+^ ions utilizing Atomic Absorption Spectroscopy (AAS). Figure 3A illustrates that the percentage of retained MnO_2_ in HFn@MnO_2_/AS nanoparticles exhibited notable stability in pH 7.4 phosphate-buffered saline (PBS) in the absence of hydrogen peroxide (H_2_O_2_), with over 90% of MnO_2_ remaining post-incubation with H_2_O_2_. This observation implies a limited catalytic activity of the MnO_2_ nano-enzyme under physiological conditions. Conversely, a noteworthy increase in the degradation rate of MnO_2_ was observed in acidic pH conditions, with the presence of H_2_O_2_ further accelerating the degradation process. This observation can be elucidated through subsequent reactions:

(1) $$\mathrm{Mn}O_{2}+H^{+}+H_{2}O_{2}\to {Mn}^{2+}+H_{2}O+O_{2}↑$$

(2) $$\mathrm{Mn}O_{2}+2H_{2}O_{2}\to 2H_{2}O+O_{2}↑$$

The oxygen production is exhibited in Figure 3B. These results suggested that the presence of MnO_2_ can effectively scavenge H_2_O_2_ and improve the oxidative stress microenvironment. Meanwhile, it was laterally corroborated that HFn could be used as a nanoreactor to successfully synthesize MnO_2_ nanoparticles. Figure 3B illustrates the manifestation of oxygen production. These findings suggest that the inclusion of MnO_2_ demonstrates an effective ability to scavenge H_2_O_2_ and enhance the oxidative stress microenvironment. Concurrently, it was laterally substantiated that HFn served as a viable nanoreactor, facilitating the successful synthesis of MnO_2_ nanoparticles.

#### 3.3.2. The Antioxidant Ability of CGPG-HFn@MnO_2_/AS Hydrogels

Subsequently, we assessed the antioxidant ability of CGPG-HFn@MnO_2_/AS hydrogels. Both unaltered hydrogels and hydrogels laden with HFn@MnO_2_ were immersed in an oxidative medium containing H_2_O_2_. The efficacy of hydrogels in counteracting ROS was ascertained by analyzing the concentration of H_2_O_2_ using the H_2_O_2_ kit (Figure 3C). As the duration of co-incubation increased, the concentration of H_2_O_2_ in the blank hydrogel group exhibited a consistent trend with no statistically significant variance. In contrast, when compared to the blank hydrogel, the hydrogel incorporating HFn@MnO_2_ nanoparticles demonstrated a notable reduction in H_2_O_2_ content following both 2 and 4 h of incubation. This observation indicated that the formulated CGPG-HFn@MnO_2_ hydrogel possessed the capacity to adeptly regulate the oxidative stress microenvironment in the context of spinal cord injury.

Furthermore, we conducted additional validation of the antioxidant properties of the synthesized HFn@MnO_2_ nanoparticles through cellular experiments. The stimulation of PC12 cells with H_2_O_2_ resulted in a noteworthy increase in intracellular ROS content. Subsequently, these cells were co-incubated with HFn@MnO_2_ nanoparticles and subsequently stained with dichlorodihydrofluorescein diacetate (DCFH-DA). As illustrated in Figure 3D,E, the fluorescence intensity of ROS in PC12 cells treated with HFn@MnO_2_ nanoparticles exhibited a significant reduction. This substantiated the assertion that HFn@MnO_2_ nanoparticles possessed the capability to effectively scavenge reactive oxygen species.

### 3.4. Cellular Uptake

The in vitro release findings revealed a sustained and gradual release behavior of AS from the hydrogel. To assess the cellular uptake of AS by PC12 cells, fluorescein isothiocyanate (FITC) was employed as a fluorescent indicator to substitute AS within the HFn carrier. The uptake of AS by PC12 cells was then examined using fluorescence microscopy. As depicted in Figure 3F, the intensity of green fluorescence exhibited a progressive augmentation with the extension of co-incubation time, signifying a notable enhancement in the effective uptake of FITC by PC12 cells. This temporal dependence of the green fluorescence intensity indirectly substantiates the hydrogel’s capacity for sustained and gradual release.

### 3.5. The Neuroprotective Effect of CGPG-HFn@MnO_2_/AS and Its Underlying Mechanism on PC12 Cells Stimulated by H_2_O_2_

**Figure 4:**
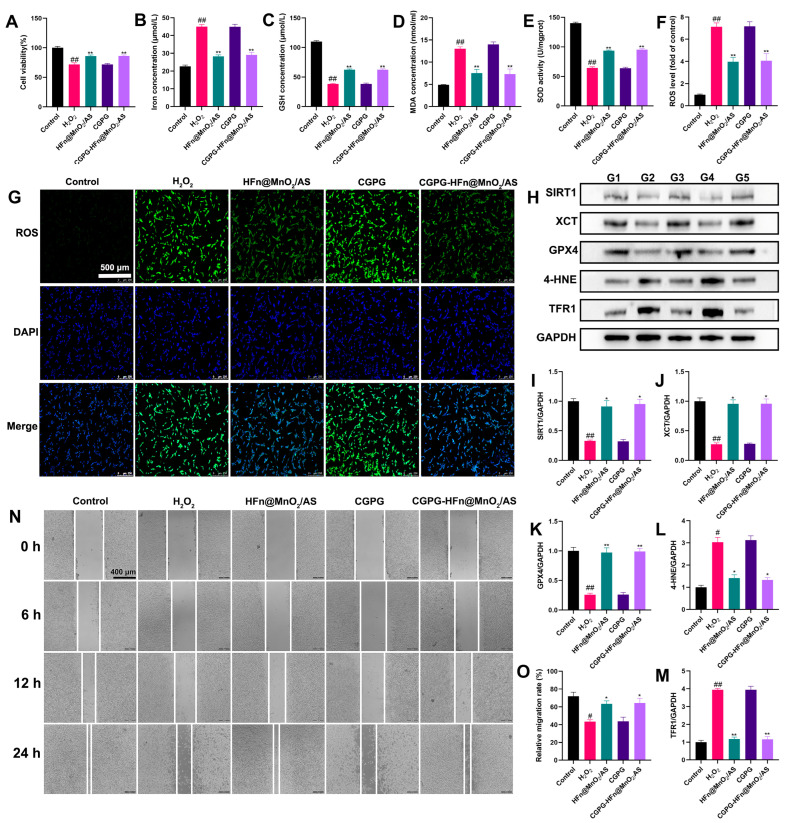
Effects of CGPG-HFn@MnO_2_/AS on H_2_O_2_-treated PC12 cells. (**A**) Cell viability was detected using the CCK-8 assay kit. (**B**) Cellular iron levels were detected using a kit. (**C**) Cellular GSH levels were detected using a kit. (**D**) Cellular MDA levels were detected using a kit. (**E**) Cellular SOD activity was detected using a kit. (**F**,**G**) Cellular ROS levels were detected using the fluorescent probe DCFH-DA. (Scale bar of **G**: 500 μm) (**H**–**M**) The protein levels of SIRT1, XCT, GPX4, 4-HNE, and TFR1 were detected by western blot assay. G1: control group. G2: H_2_O_2_ group. G3: HFn@MnO_2_/AS group. G4:CGPG group. G5: CGPG-HFn@MnO_2_/AS. (**N**,**O**) Cell migration was evaluated using the wound healing assay. (Scale bar of **N**: 400 μm)Statistical analyses were performed using a one-way analysis of variance (ANOVA), followed by Tukey’s post-hoc test (n ≥ 3). # *p* < 0.05 and ## *p* < 0.01 vs. the control group; * *p* < 0.05 and ** *p* < 0.01 vs. the H_2_O_2_ group.

The generation of excess ROS is a crucial factor affecting neuronal function and mediating neuronal death after SCI [27]. H_2_O_2_ stimulation produces a large number of ROS, induces oxidative stress injury, and accelerates neuron death in PC12 cells [28]. To investigate the neuroprotective effect of CGPG-HFn@MnO_2_/AS on PC12 cells stimulated by H_2_O_2_, CCK-8 assays were used to measure cell viability. As shown in Figure 4A, H_2_O_2_ stimulation significantly decreased the viability of PC12 cells, while pretreatment with HFn@MnO_2_/AS or CGPG-HFn@MnO_2_/AS significantly increased the cell viability as compared to the H_2_O_2_ group. CGPG-HFn@MnO_2_/AS reduced cell death caused by the H_2_O_2_ challenge. Suppressing oxidative stress attenuated mitochondrial injury and blocked signaling pathways associated with ferroptosis in HT22 neuron cells [29]. Oxidative stress-induced ferroptosis has been reported as an important factor in drug-induced PC12 cell death [30]. The levels of Iron, GSH, MDA, and ROS and the activity of SOD are considered key indicators of oxidative stress and ferroptosis. As shown in Figure 4B–E, pretreatment with HFn@MnO_2_/AS or CGPG-HFn@MnO_2_/AS significantly decreased the concentration of iron; MDA, on the contrary, increased the GSH levels and SOD activity in H_2_O_2_-stimulated cells by kit detection. Additionally, the fluorescent probe DCFH-DA was used to detect ROS production in vitro. As shown in Figure 4F,G, H_2_O_2_ stimulation promoted the release of a large number of ROS, while pretreatment with HFn@MnO_2_/AS or CGPG-HFn@MnO_2_/AS visibly reversed the levels of ROS. Additionally, ferroptosis-related proteins were examined by western blot. As shown in Figure 4H–M, the expression of XCT and GPX4 significantly decreased, while the expression of 4-HNE and TFR1 significantly increased after H_2_O_2_ stimulation. PC12 cells pretreated with HFn@MnO_2_/AS or CGPG-HFn@MnO_2_/AS exhibited a significant reverse of the expression of these proteins. Combined with these results and previous studies, we hold the opinion that CGPG-HFn@MnO_2_/AS could alleviate oxidative stress-induced ferroptosis in nerve cells. Previous studies have shown that inhibition of SIRT1 could exacerbate oxidative damage, neuroinflammation, and neuronal apoptosis, while activation of SIRT1-mediated neuroprotective effects promotes mitochondrial function and cell survival [31,32]. As a class III histone deacetylase, SIRT1 has demonstrated an important ability to extend cellular lifespan by regulating genomic stability, neuronal growth, energy metabolism, oxidative stress, and so on [33]. In our results, H_2_O_2_-stimulated cells exhibited a significant decrease in SIRT1 expression, while pretreatment with HFn@MnO_2_/AS or CGPG-HFn@MnO_2_/AS clearly increased the protein level of SIRT1. These findings indicated that CGPG-HFn@MnO_2_/AS inhibited oxidative stress-induced ferroptosis by regulating SIRT1 signaling in vitro. Next, a wound healing assay was further used to evaluate the neural repair effect of CGPG-HFn@MnO_2_/AS. Wound healing experiments involve cell migration, and promoting cell migration is conducive to neuron repair. As shown in Figure 4N,O, H_2_O_2_ stimulation significantly inhibited scratch wound closure of PC12 cells, and pretreatment with HFn@MnO_2_/AS or CGPG-HFn@MnO_2_/AS showed a significant rate of cell migration compared to the H_2_O_2_ group. The results demonstrated that CGPG-HFn@MnO_2_/AS is beneficial to neuronal repair after oxidative stress injury. Therefore, these in vitro experiments demonstrate the therapeutic significance of CGPG-HFn@MnO_2_/AS in neuronal injury.

### 3.6. The Repair Effect and Mechanism of CGPG-HFn@MnO_2_/AS on Spinal Cord Injury in Rats

**Figure 5:**
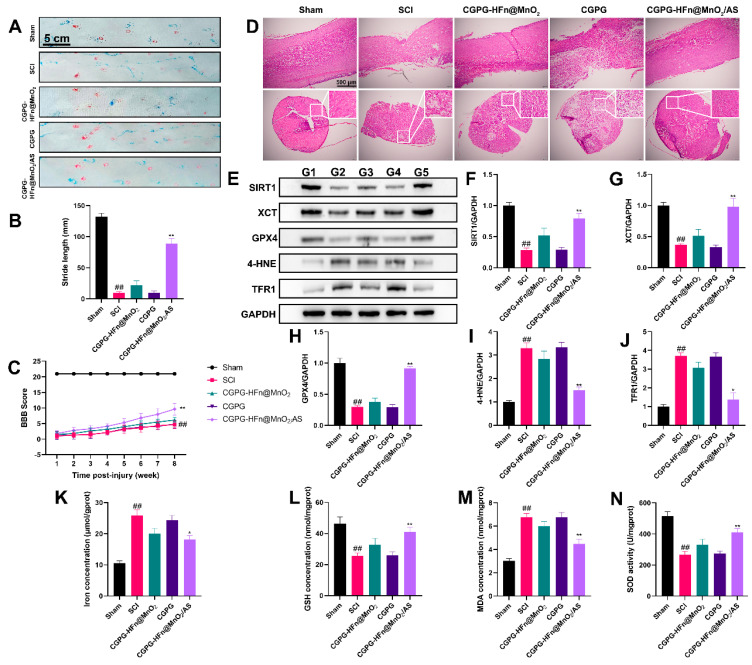
Effects of CGPG-HFn@MnO_2_/AS on rats undergoing SCI. (**A**,**B**) The hind-limb motor function recovery of SCI rats was evaluated using footprint analysis. ## *p* < 0.01 vs. the control group; ** *p* < 0.01 vs. the SCI group. (**C**) The degree of hind-limb recovery was evaluated by the BBB score. ## *p* < 0.01 vs. the sham group; ** *p* < 0.01 vs. the SCI group. (**D**) The histological lesion was evaluated using H&E staining. (**E**–**J**) The protein levels of SIRT1, XCT, GPX4, 4-HNE, and TFR1 were detected using a western blot assay. G1: sham group. G2: SCI group. G3: CGPG-HFn@MnO_2_/AS group. G4:CGPG group. G5: CGPG-HFn@MnO_2_/AS. (**K**) The level of iron in the spinal cord was detected using a kit. (**L**) The level of GSH in the spinal cord was detected using a kit. (**M**) The level of MDA in the spinal cord was detected using a kit. (**N**) The activity of SOD in the spinal cord was detected using a kit. Statistical analyses were performed using a one-way analysis of variance (ANOVA), followed by Tukey’s post-hoc test (n ≥ 3). ## *p* < 0.01 vs. the sham group; * *p* < 0.05 and ** *p* < 0.01 vs. the SCI group.

To further clarify the effects of CGPG-HFn@MnO_2_/AS on spinal cord injury, a rat model of SCI was established to explore the repair effects and mechanisms of the drugs. As shown in Figure 5A–C, the BBB exercise scale and footprint analysis were used to evaluate motor function in rats. Rats who suffered from SCI scored lower points than the sham group in the BBB motor function evaluation; treatment with CGPG-HFn@MnO_2_/AS significantly reversed the motor function of SCI rats. The typical gait images of rats showed that rats in the model group exhibited severe hind-limb paralysis. The stride length of SCI rats was significantly reduced as compared to the sham group, while treatment with CGPG-HFn@MnO_2_/AS significantly improved the motor function of the hind limbs, and the results were consistent with the BBB evaluation. As shown in Figure 5D, histology was used to evaluate the repair capability of CGPG-HFn@MnO_2_/AS in injured spinal cords. The results of HE staining showed that the tissue morphology in the SCI group was changed, and the number of intact neurons in the SCI group was reduced as compared to the sham group, while treatment with CGPG-HFn@MnO_2_/AS inhibited the loss of neurons and mitigated tissue damage. These results implicated that CGPG-HFn@MnO_2_/AS promotes the regeneration of SCI and possesses neuroprotective effects in SCI rats.

There is a growing body of evidence that ferroptosis contributes to the collapse of the blood-spinal cord barrier and participates in the pathogenesis of SCI [13,34]. SIRT1, as a mammalian NAD(+)-dependent protein deacetylase, has been proven to regulate the ferroptosis process [35,36]. Activation of SIRT1 ameliorated ferroptosis and neuronal damage in both in vivo and in vitro experiments [37]. SIRT1 deficiency exacerbated oxidative stress injury and ferroptosis [38]. Therefore, we determined the levels of ferroptosis-related proteins in the spinal cords of animals. As shown in Figure 5E–J, the expression of the proteins SIRT1, XCT, and GPX4 in the SCI group was significantly decreased, while the expression of the proteins 4-HNE and TFR1 was increased. Treatment with CGPG-HFn@MnO_2_/AS significantly inhibited the changes in the expression of these proteins. Our results were supported by the fact that SIRT1 activation induced by the agonist SRT1720 decreased the endothelial ROS production, attenuated the oxidative stress of neurons, protected the function of the blood-spinal cord barrier [39]. Ferroptosis is characterized by an iron-dependent cell death pathway, which is involved in glutamate accumulation, the release of ROS, REDOX imbalance, and lipid peroxidation [40,41]. As shown in Figure 5K–N, following SCI, the rats exhibited significant increases in iron accumulation and MDA levels and decreases in GSH levels and SOD activity. Treatment with CGPG-HFn@MnO_2_/AS dramatically reversed these changes. Based on these findings, we hold the opinion that CGPG-HFn@MnO_2_/AS could promote the recovery of spinal neuron function and maintain brain homeostasis after SCI by ameliorating oxidative stress injury and inhibiting ferroptosis via SIRT1 signaling. In the future, CGPG-HFn@MnO_2_/AS is a promising candidate for the treatment of SCI.

### 3.7. Safety Analysis

**Figure 6:**
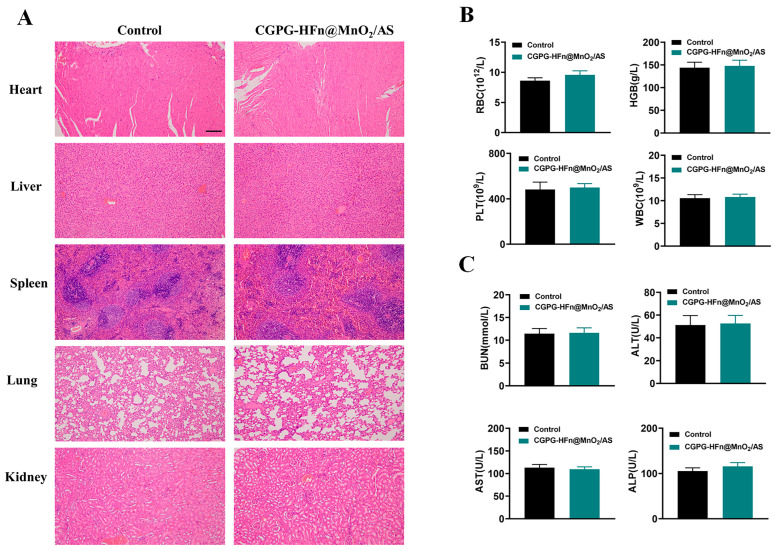
Safety analysis of CGPG-HFn@MnO_2_/AS hydrogels. (**A**) H&E staining images of different organs (heart, liver, spleen, lung, and kidney) in healthy SD rats after administration (n = 3) (Scale bar: 200 μm). (**B**) Hematology analysis of RBCs, HGBs, PLTs, and WBCs in different groups (n = 3). (**C**) Serum biochemical analysis of BUN, ALT, AST, and ALP in different groups (n = 3).

To comprehensively evaluate the in vivo safety of CGPG-HFn@MnO_2_/AS hydrogels, H&E staining was performed on the heart, liver, spleen, kidney, and lung tissues obtained from healthy SD rats after administration of nanoparticles. Figure 6A shows the comparison of the H&E staining results between rats in the control and treatment groups. It was worth noting that no obvious pathological changes in morphology were found in the examination of all organs. In addition, blood samples were collected for hematological and serum biochemical analysis. As shown in Figure 6B, the results of hematological analysis showed that there were no significant differences in the concentrations of WBCs, RBCs, HGBs, and PLTs between the groups, all of which were within the normal physiological range. In addition, the biochemical analysis in Figure 6C showed that the levels of BUN, AST, ALT, and ALP in all groups were within the normal range, and there were no significant differences between the treatment groups. Therefore, the above results indicated that CGPG-HFn@MnO_2_/AS hydrogels had good safety.

## 4. Conclusions

In summary, this study successfully synthesized HFn as a nanoreactor, facilitating the generation of MnO_2_ nanoparticles within its inner cavity while concurrently encapsulating AS. The resulting composite was then dispersed within CGPG hydrogels endowed with temperature-sensitive properties. The formulated CGPG-HFn@MnO_2_/AS hydrogel demonstrated notable efficacy in scavenging ROS, both in vivo and in vitro, thereby mitigating neuronal cell damage and apoptosis within the oxidative stress microenvironment post-SCI. Simultaneously, the proficient delivery of AS inhibited oxidative stress-induced ferroptosis through the regulation of SIRT1 signaling, consequently promoting neuronal cell migration and repair. The outcomes further indicated that CGPG-HFn@MnO_2_/AS treatment significantly reversed motor dysfunction and facilitated the repair of neuronal cells at the injury site in SCI rats. Thus, the engineered hydrogel, incorporating MnO_2_ and AS, exhibited a comprehensive capacity to modulate the pathological microenvironment, presenting a promising therapeutic avenue for CNS diseases.

## Data Availability

The raw data supporting the conclusions of this article will be made available by the authors on request.
